# Implementation of the HUGO™ RAS system for robot-assisted radical prostatectomy: a single-center comparison with the da Vinci Xi^®^ system

**DOI:** 10.1007/s00345-026-06870-8

**Published:** 2026-10-28

**Authors:** Anton Moderegger, Reinhard Vonthein, Hendrik Eike Fender, Alexander Stephan Reese, Friederike Praus, Marie Christine Roesch, Axel S. Merseburger, Daniar Osmonov

**Affiliations:** 1 Department of Urology, University Medical Center Schleswig-Holstein, Campus Luebeck, Ratzeburger Allee 160, 23538 Luebeck, Germany; 2 Institute of Medical Biometry and Statistics, University of Luebeck, Luebeck, Germany

**Keywords:** Robot-assisted radical prostatectomy, HUGO™ RAS system, Da Vinci Xi^®^, Propensity score, Overlap weighting, Surgical outcomes

## Abstract

**Purpose:**

To compare early perioperative and pathological outcomes between the HUGO™ RAS and da Vinci Xi^®^ robotic platforms for robot-assisted radical prostatectomy (RARP) at a single center.

**Methods:**

We retrospectively analyzed 107 consecutive patients who underwent RARP (50 HUGO™, 57 da Vinci™) between April 2024 and December 2025, performed by two surgeons who both used both systems. Outcome-specific propensity score models with overlap weighting were used to estimate adjusted associations between platform and operative duration, length of hospital stay, anastomotic integrity, positive surgical margins, lymph node yield, complications, and achieved nerve sparing.

**Results:**

Baseline characteristics were broadly comparable, although the HUGO™ cohort included a higher proportion of high-risk D’Amico tumors. Operative duration was longer with HUGO™ in the unadjusted analysis (211 vs. 193 min, *p* = 0.036); after overlap weighting and covariate adjustment, the difference was 14.6 min (95% CI -3.0 to 32.2) and not statistically significant. No statistically significant adjusted differences were observed between platforms for length of hospital stay, anastomotic integrity, positive surgical margins, lymph node yield, complications, or achieved nerve sparing.

**Conclusion:**

In this early single-center experience, RARP performed with the HUGO™ RAS system showed no statistically significant differences in early perioperative and pathological outcomes compared with the established da Vinci Xi^®^ platform after adjustment for measured confounders. Larger prospective multicenter studies with longer follow-up are needed to confirm these findings and to evaluate long-term oncological and functional outcomes.

**Supplementary Information:**

The online version contains supplementary material available at https://doi.org/10.1007/s00345-026-06870-8.

## Introduction

Radical prostatectomy (RP) remains a cornerstone in the management of localized prostate cancer. Over the past two decades, robot-assisted radical prostatectomy (RARP) has become the predominant surgical approach in many healthcare systems, largely replacing open surgery due to its minimally invasive nature and favorable perioperative outcomes [5, 13, 15].

Since its introduction in 2000, the da Vinci™ surgical system (Intuitive Surgical, Sunnyvale, CA, USA) has dominated the field of robotic surgery, with early clinical reports demonstrating the feasibility of RARP [5]. However, the widespread adoption of robotic platforms has been associated with substantial costs related to acquisition, maintenance, and disposable instruments [4].

Following the expiration of key patents around 2019, several new robotic platforms have entered the market. Among these, the HUGO™ RAS system (Medtronic, Minneapolis, MN, USA) has emerged as a novel alternative. High-quality evidence, particularly regarding long-term oncological and functional outcomes, remains limited, and further comparative studies are warranted to define the role of emerging robotic systems in urologic surgery [2, 16].

Early clinical series [1, 17] and comparative analyses have demonstrated its feasibility and safety, with perioperative and early functional outcomes comparable to those of established robotic platforms in a systematic review [16]. That review was not followed by meta-analyses. Therefore, we performed a retrospective comparative analysis of our initial experience with the HUGO™ RAS system and a contemporary da Vinci Xi^®^ cohort treated at the same institution.

## Materials and methods

The HUGO™ RAS system (Medtronic, Minneapolis, MN, USA) has been in routine clinical use at our institution since April 2024, whereas the da Vinci Xi^®^ surgical system (Intuitive Surgical, Sunnyvale, CA, USA) has been routinely used since 2017. Both robotic platforms were available during the study period and were considered clinically equivalent. No platform-specific exclusion criteria were applied. Choice of surgical platform was not randomized or blinded. Allocation was influenced by operating-room scheduling, as HUGO™ and da Vinci™ procedures were predominantly performed on different standard operating days (Mondays and Thursdays, respectively), and by case-by-case surgeon judgment, with a preference for the da Vinci Xi^®^ in anatomically borderline cases in which nerve-sparing was planned but the procedure was expected to be technically demanding.

We retrospectively analyzed consecutive patients who underwent RARP at the Department of Urology, University Hospital Schleswig-Holstein, Campus Luebeck, between April 2024 and December 2025. Data were retrospectively retrieved from the ORBIS^®^ AQURA hospital information and OR-management system. Fifty patients underwent RARP using the HUGO™ RAS system and 57 patients underwent RARP using the da Vinci Xi^®^ system during the same period. All procedures were performed by two robotic surgeons, each with more than 250 da Vinci™ RARPs performed before the study period, who both used both systems. Neither surgeon had performed clinical HUGO™ procedures before the study period; prior HUGO™ exposure was limited to simulator-based and proctored training. Technical success was defined as completion of the procedure with the robotic platform used. All HUGO™ procedures attempted during the study period were included in the denominator for technical success, including one case in which docking failure required conversion to open surgery; because no robotic steps were performed in this case, it was not included in the perioperative and pathological outcome analyses.

All RARPs were carried out using a transperitoneal approach. Port placement and docking were performed according to the respective manufacturer’s recommendations. The decision to perform nerve-sparing surgery was based on comprehensive preoperative counseling and individual oncological risk assessment, considering patient preference, prostate-specific antigen (PSA) level, biopsy findings, magnetic resonance imaging (MRI) results, and baseline erectile function. Intraoperative findings were additionally considered during final decision-making.

Intraoperative frozen-section analysis was routinely performed at the bladder neck and urethral margins before vesicourethral anastomosis. In cases of nerve-sparing surgery, frozen-section analysis of the neurovascular bundles was additionally performed when indicated.

Preoperative patient characteristics included age, body mass index (BMI), American Society of Anesthesiologists (ASA) physical status classification, PSA level, prostate volume, preoperative biopsy Gleason score, planned nerve-sparing procedure, and operating surgeon.

The primary perioperative outcomes were operative duration, length of hospital stay, anastomotic integrity at the first postoperative cystogram, and postoperative complications. The extent of planned and achieved nerve-sparing surgery was analyzed descriptively because complete separation precluded adjusted regression analysis.

Retrograde cystography was performed by the Department of Urology (not scheduled on weekends or public holidays), using retrograde instillation of 100 mL contrast medium followed by 100 mL water, with anastomotic integrity confirmed fluoroscopically in two planes during bladder filling and after voiding; any visible contrast extravasation, however minor, was classified as a non-intact anastomosis, and catheter removal was performed only after a negative (intact) cystogram. Cystography was performed predominantly on postoperative day 4 for HUGO™ cases (30/50, 60%) and postoperative day 5 for da Vinci™ cases (50/57, 88%), reflecting the platforms’ differing standard operating weekdays.

Because all tumors were clinically non-palpable (cT1c), D’Amico risk groups were assigned using preoperative PSA level and biopsy Gleason score only [8].

Continuous variables are presented as mean ± standard deviation (SD) and median or median with interquartile range, as appropriate. Categorical variables are presented as frequencies and percentages. Between-group comparisons were performed using the Wilcoxon rank-sum test for continuous variables and Fisher’s exact test for categorical variables. Statistical significance was defined as a two-sided p-value < 0.05. Because multiple outcomes were compared without adjustment for multiplicity, these unadjusted p-values are reported as descriptive/exploratory statistics and should be interpreted alongside the overlap-weighted, covariate-adjusted estimates presented below.

To account for potential baseline differences between treatment groups, outcome-specific propensity score analyses were performed. Covariates for each propensity score model were selected a priori based on established predictors of the respective outcome reported in previous studies and contemporary prostate cancer guidelines [6, 7, 8, 14, 18].

Propensity scores were estimated using logistic regression, and overlap weighting was applied to estimate the average treatment effect in the overlap population (ATO), corresponding to patients who could plausibly have undergone surgery with either robotic platform. By construction, overlap weighting achieves exact balance for the covariates included in the propensity score model. Numerical standardized mean differences and effective sample sizes before and after weighting are reported for each outcome-specific model in Supplementary Table S3; no model showed evidence of highly influential individual weights beyond the small number of extreme-propensity cases already discussed in the Limitations. Expressed relative to a weight of 1 in an unweighted analysis, weights ranged from 0.23 to 1.83 across all outcome-specific models (Supplementary Table S3). Propensity score distributions are illustrated in Supplementary Figure S2, whereas the resulting covariate balance before and after weighting is shown in Supplementary Figure S1.

Outcome models were fitted using overlap weights to reduce measured confounding between treatment groups and were additionally adjusted for the covariates included in the corresponding propensity score model for estimation of adjusted associations. This additional adjustment was applied to explain residual between-patient variability in the balanced, weighted sample and thereby increase the precision of the estimates. Because platform allocation was not randomized and was informed in part by anticipated case complexity, these estimates are best interpreted as adjusted associations rather than causal treatment effects, and residual confounding by unmeasured factors cannot be excluded. The outcome-specific covariates included in each propensity score model are summarized in Supplementary Table S1 and were selected according to their anticipated clinical relevance for the respective endpoint. For the endpoint of achieved nerve sparing, planned nerve sparing was included in the propensity score model but omitted from the weighted outcome model because of its near-deterministic association with the outcome. Outcome models were chosen according to the distribution and measurement scale of each endpoint. For continuous outcomes, model assumptions were evaluated using Box–Cox transformation analysis and residual diagnostics. Operative duration was analyzed using weighted linear regression, whereas length of hospital stay was analyzed using an inverse Gaussian generalized linear model with an inverse link. Anastomotic integrity at the first postoperative cystogram, positive surgical margins, and postoperative complications were analyzed using weighted logistic regression models. Lymph node yield was analyzed using a weighted negative binomial regression model because the count data exhibited overdispersion. Adjusted treatment effects are reported as regression coefficients (β), odds ratios (OR), or incidence rate ratios (IRR) with corresponding 95% confidence intervals (CI), as appropriate. To facilitate comparison of platform- and surgeon-related effects across the investigated endpoints, the adjusted effect estimates of both variables from the final outcome-specific regression models are summarized in Supplementary Table S2.

To facilitate clinical interpretation, marginal expected treatment effects were estimated using standardized model-based predictions. These were obtained by assigning all patients hypothetically to either the HUGO™ or the da Vinci™ platform while retaining their observed covariate values and averaging the resulting model-based predictions across the study population. This approach estimates the expected outcomes if all patients eligible for both robotic platforms had undergone surgery with either platform.

To characterize the institutional learning curve, operative time for the 50 HUGO™ cases was additionally modeled as a function of surgery date and of consecutive case number using simple linear regression, and the unadjusted comparison of operative time was repeated after excluding the first 10 and 15 HUGO™ cases as a sensitivity analysis. All statistical analyses were performed using R version 4.6.0 (R Foundation for Statistical Computing, Vienna, Austria) within RStudio version 2026.05.0 + 218.

Institutional Review Board approval was obtained from the Ethics Committee of the University of Luebeck prior to data collection (reference number 2025 − 214).

Generative artificial intelligence (ChatGPT, OpenAI, San Francisco, CA, USA) was used to support statistical code development, language editing, and manuscript preparation. All statistical analyses, interpretations, and manuscript content were reviewed and verified by the authors, including a co-author biometrician, who take full responsibility for the accuracy and integrity of the work.

## Results

### Baseline characteristics

Baseline patient characteristics are summarized in Table 1.

**Table 1:** Baseline characteristics according to robotic platform

Characteristic	HUGO™ *N* = 50	da Vinci™ *N* = 57	*P*
Age (yr)	65.7 (± 6.4); 65	65 (± 5.6); 66	0.550
Body mass index (kg/m²)	28.4 (± 4.2); 27.5	27.2 (± 3.1); 27.5	0.342
ASA			0.070
1	2 (4%)	10 (17.5%)	
2	37 (74%)	39 (68.4%)	
3	11 (22%)	8 (14%)	
Preoperative PSA (ng/ml)	9 (5.9–13.9)	8.3 (5.6–11)	0.221
Preoperative Gleason score			0.695
6	2 (4%)	3 (5.3%)	
7a	21 (42%)	31 (54.4%)	
7b	16 (32%)	13 (22.8%)	
8	3 (6%)	2 (3.5%)	
9	7 (14%)	8 (14%)	
10	1 (2%)	0 (0%)	
D’Amico risk group			0.125
Low	1 (2%)	3 (5.3%)	
Intermediate	31 (62%)	43 (75.4%)	
High	18 (36%)	11 (19.3%)	
Size of prostate (ml)	47.3 (± 22.4); 41	41.4 (± 15.6); 39	0.301
Surgeon			0.556
A	27 (54%)	35 (61.4%)	
B	23 (46%)	22 (38.6%)	

Values are presented as mean (± SD); median, median (Q1–Q3), or n (%).

P values were calculated using Wilcoxon rank-sum tests for continuous variables and Fisher’s exact tests for categorical variables

Overall, the two cohorts were well balanced with respect to demographic and preoperative clinical characteristics. Patients in the HUGO™ cohort tended to have a higher comorbidity burden, with a greater proportion of ASA III patients (22% vs. 14%), although this difference did not reach statistical significance (*p* = 0.070). Likewise, patients treated with the HUGO™ system more frequently belonged to the high-risk D’Amico group (36% vs. 19%), whereas intermediate-risk disease was more common in the da Vinci™ cohort (75% vs. 62%); however, this difference was not statistically significant (*p* = 0.125). No significant differences were observed regarding age, body mass index, preoperative PSA level, Gleason score distribution, prostate volume, or surgeon distribution.

### Perioperative and postoperative outcomes

Perioperative and postoperative outcomes are summarized in Table 2.

**Table 2:** Perioperative and postoperative outcomes according to robotic platform

Outcome	HUGO™ *N* = 50	da Vinci™ *N* = 57	*p*-value
Operative duration (min) (mean ± SD; median)	211 (± 43); 204	193 (± 45); 188	0.036
Positive intraoperative frozen section, n (%)	2 (4%)	6 (11%)	0.293
Pathological T stage, n (%)			0.956
pT2a	3 (6%)	2 (4%)	
pT2b	0 (0%)	0 (0%)	
pT2c	22 (44%)	25 (44%)	
pT3a	16 (32%)	19 (33%)	
pT3b	9 (18%)	11 (19%)	
Positive surgical margins (R1), n (%)	18 (36%)	22 (39%)	0.843
Number of lymph nodes (mean ± SD; median)	9.8 (± 4.8); 9	10.4 (± 6.9); 9	0.763
Patients with positive lymph nodes, n (%)	0 (0%)	6 (11%)	0.029
Clavien-Dindo, n (%)			0.849
0	41 (82%)	44 (77%)	
1	5 (10%)	7 (12%)	
2	0 (0%)	2 (4%)	
3a	3 (6%)	3 (5%)	
3b	1 (2%)	1 (2%)	
4/5	0 (0%)	0 (0%)	
Hospital stay (days) (mean ± SD; median)	6.6 (± 2.1); 6	6.5 (± 2); 6	0.993
Catheterization time (days) (mean ± SD; median)	12.3 (± 12.3); 5	11.5 (± 12.2); 5	0.368
Anastomotic integrity at first cystogram, n (%)	33 (66%)	39 (68%)	0.838

Values are presented as n (%) or mean (± SD); median

Percentages for intraoperative frozen section are based on evaluable cases only; four HUGO™ cases had no evaluable frozen-section result. P-values were calculated using Wilcoxon rank-sum tests for continuous variables and Fisher’s exact tests for categorical variables

Operative time was significantly longer in the HUGO™ cohort in the unadjusted analysis. Operative time with HUGO™ decreased by 0.79 min per consecutive case (95% CI −1.62 to 0.04, *p* = 0.063), corresponding to approximately 39 min across the 50-case cohort (Supplementary Figure S4). In a sensitivity analysis excluding the first 10 HUGO™ cases, the unadjusted difference in mean operative time decreased from 17.7 min (210.9 vs. 193.2 min; *p* = 0.036) to 11.9 min (205.1 vs. 193.2 min; *p* = 0.097); excluding the first 15 cases did not reduce it further (12.2 min; *p* = 0.116). Apart from operative duration, no significant differences were observed between robotic platforms regarding pathological stage, positive surgical margins, lymph node yield, length of hospital stay, anastomotic integrity at the first postoperative cystogram, or overall postoperative complications. Positive surgical margin rates stratified by pathological stage did not differ significantly between platforms, although subgroup sizes were small: 24.0% (6/25) vs. 29.6% (8/27) for pT2 disease (OR 0.75, 95% CI 0.18–3.04, *p* = 0.759) and 48.0% (12/25) vs. 46.7% (14/30) for pT3 disease (OR 1.05, 95% CI 0.32–3.47, *p* = 1.000) in the HUGO™ and da Vinci™ groups, respectively.

Positive lymph nodes were identified exclusively in the da Vinci™ cohort (11% vs. 0%, *p* = 0.029). Because no positive lymph nodes occurred in the HUGO™ cohort, an adjusted regression analysis for this endpoint was not feasible (complete separation). Given the small number of events and the higher proportion of high-risk disease in the HUGO™ cohort, this unadjusted finding most likely reflects case-mix and sampling variability rather than a true platform-related difference and should be interpreted as a case-selection effect rather than a positive finding.

Overall postoperative morbidity was low in both groups. Most complications were minor (Clavien–Dindo grades 0–1). In the HUGO™ cohort, three patients required Clavien–Dindo grade 3a interventions (two lymphocele drainages and one mono-J catheter placement under local anesthesia), while one patient underwent emergency laparotomy because of postoperative bleeding (grade 3b). In the da Vinci™ cohort, two patients required readmission for intravenous antibiotic treatment (grade 2), three underwent grade 3a interventions requiring cystoscopic urinary catheter placement, ultrasound-guided drainage of a pelvic fluid collection, and percutaneous nephrostomy placement, respectively. One further patient developed a Clavien–Dindo grade 3b complication requiring mono-J ureteral stent placement following bladder perforation.

Two notable technical events occurred during use of the HUGO™ platform. One robotic arm malfunction during vesicourethral anastomosis prolonged operative time without clinical consequences. In another case, docking failure necessitated conversion to open surgery. This case was excluded from this dataset. As no part of the procedure was completed with the HUGO™ system in this case, it is not among the 50 HUGO™ patients analyzed in this study. Including this case, the HUGO™ system was used in a total of 51 RARP attempts during the study period, of which 50 (98.0%) were completed robotically and 1 (2.0%) required conversion to open surgery because of docking failure before any portion of the procedure could be performed robotically. This technical failure is reported here as a platform-related outcome; because no robotic steps were performed, the case could not be meaningfully included in the perioperative and pathological outcome analyses, which are therefore based on the 50 patients in whom the robotic procedure was completed.

### Nerve-sparing surgery

Planned and achieved outcomes are summarized in Table 3.

**Table 3:** Planned and achieved nerve sparing according to robotic platform

Outcome	HUGO™	da Vinci™	*P*
Nerve sparing planned	21 (42%)	36 (63.2%)	0.034¹
Bilateral nerve sparing achieved	21 (42%)	29 (50.9%)	0.438¹
Among patients with planned nerve sparing			
No nerve sparing achieved	0 (0%)	5 (13.9%)	
Unilateral nerve sparing	0 (0%)	2 (5.6%)	
Bilateral nerve sparing	21 (100%)	29 (80.6%)	0.034²

¹ Fisher’s exact test comparing the frequency of nerve sparing

² Wilcoxon rank-sum test comparing the ordinal extent of achieved nerve-sparing (none, unilateral, bilateral) among patients with planned nerve-sparing surgery

Nerve-sparing surgery was planned significantly more often in the da Vinci™ cohort than in the HUGO™ cohort (63.2% vs. 42.0%, *p* = 0.034). Among patients in whom nerve-sparing surgery had been planned, bilateral nerve preservation was achieved in all 21 patients (100%) treated with the HUGO™ system and in 29 of 36 patients (80.6%) treated with the da Vinci™ system; two da Vinci™ patients (5.6%) had unilateral and five (13.9%) no nerve preservation (*p* = 0.034). The unconditional rates of bilateral nerve sparing were 42.0% with HUGO™ and 50.9% with da Vinci™ (*p* = 0.438).

Because complete separation occurred, adjusted logistic regression analysis of deviation from the planned nerve-sparing procedure was not performed. However, achieved nerve sparing (unilateral or bilateral vs. none) was analyzed using an outcome-specific overlap-weighted logistic regression model. After overlap weighting and adjustment for surgeon, D’Amico risk group, and prostate volume, no statistically significant difference in achieved nerve sparing was observed between the HUGO™ and da Vinci™ platforms (OR 1.60, 95% CI 0.44–6.27; *p* = 0.481).

### Outcome-specific propensity score analyses

Propensity score distributions demonstrated substantial overlap between treatment groups for all investigated outcomes (Supplementary Figure S2). A small number of patients exhibited extreme propensity scores close to 0 or 1, reflecting treatment characteristics strongly associated with one robotic platform. By design, these observations received only minimal overlap weights and therefore contributed little to the estimation of treatment effects. Outcome-specific propensity score model estimates are summarized in Table 4.

**Table 4:** Outcome-specific propensity score model estimates (β, OR, IRR, 95% CI, p)

Outcome	Estimate	95% CI	*P*
Operative duration	β = +14.6 min	−3.0 to 32.2	0.104
Length of hospital stay	β = −0.003	−0.022 to 0.015	0.720
Anastomotic integrity at first cystogram	OR = 1.22	0.35 to 4.36	0.755
Positive surgical margins	OR = 0.69	0.20 to 2.35	0.555
Lymph node yield	IRR = 0.87	0.65 to 1.17	0.359
Complications (Clavien-Dindo > 0)	OR = 0.83	0.18 to 3.60	0.803
Nerve sparing achieved	OR = 1.60	0.44 to 6.27	0.481

Estimates are derived from outcome-specific overlap-weighted regression models with additional covariate adjustment

*β* Regression coefficient; *OR* odds ratio; *IRR* Incidence rate ratio; *CI* Confidence interval

For length of hospital stay, β is shown on the inverse link scale

After overlap weighting and additional covariate adjustment, the initially observed difference in operative duration was no longer statistically significant. Likewise, no significant differences between the HUGO™ and da Vinci™ platforms were observed for length of hospital stay, anastomotic integrity at the first postoperative cystogram, positive surgical margins, lymph node yield, or postoperative complications.

The standardized model-based predictions are summarized in Table 5.

**Table 5:** Model-based adjusted outcome estimates

Outcome	Adjusted HUGO™	Adjusted da Vinci™	Adjusted absolute difference
Operative duration	208.8 min	194.2 min	+ 14.6 min
Length of hospital stay	6.6 days	6.4 days	+ 0.1 days
Anastomotic integrity at first cystogram	69.4%	65.4%	+ 4.0% points
Positive surgical margins	34.2%	41.8%	−7.6% points
Lymph node yield	9.6 nodes	11.0 nodes	−1.4 nodes
Complications (Clavien-Dindo > 0)	18.5%	21.3%	−2.8% points
Nerve sparing achieved	52.4%	43.5%	+ 8.9% points

Values represent standardized model-based predictions obtained from the outcome-specific overlap-weighted regression models with additional covariate adjustment (stratified-adjusted estimation). Predicted outcomes were obtained by assigning all patients hypothetically to either the HUGO™ or the da Vinci™ platform while retaining their observed covariate values, and averaging the resulting model-based predictions across the study population. Adjusted absolute differences represent HUGO™ minus da Vinci™

Across all investigated endpoints, the adjusted absolute differences between the HUGO™ and da Vinci™ platforms were small, thereby supporting the findings of the overlap-weighted regression analyses. For example, the model-based estimate for positive surgical margins in the da Vinci™ cohort (41.8%) closely approximated the directly observed rate (39%; Table 2), illustrating that these standardized predictions are consistent with, but conceptually distinct from, the observed outcome frequencies.

Comparison of adjusted platform and surgeon effects is provided in Supplementary Table S2. Surgeon-related effects were not statistically significant for operative duration, length of hospital stay, anastomotic integrity at the first postoperative cystogram, positive surgical margins, or postoperative complications. In contrast, surgeon B demonstrated a significantly higher lymph node yield (IRR 1.50, 95% CI 1.11–2.04; *p* = 0.008), whereas no significant platform effect was observed for this endpoint.

## Discussion

In this single-center retrospective comparison, RARP performed with the HUGO™ RAS system demonstrated no statistically significant differences in early perioperative and pathological outcomes compared with those achieved with the established da Vinci Xi^®^ platform. Although patients in the HUGO™ cohort tended to present with a slightly less favorable baseline risk profile, including a higher proportion of ASA III patients and a greater proportion of high-risk D’Amico tumors, baseline characteristics were otherwise comparable between cohorts. No statistically significant differences were observed regarding positive surgical margins, lymph node yield, postoperative complications, duration of hospitalization, or duration of catheterization in our short case series. These findings were supported using outcome-specific overlap-weighted propensity score analyses with adjustment for clinically relevant confounders.

Operative duration was approximately three hours in both groups (208.8 vs. 194.2 min). The standardized model-based predictions indicated that operative duration was 14.6 min longer with the HUGO™ platform (95% CI −3.0 to 32.2 min), a difference that was not statistically significant. Although such a difference may have economic implications in terms of operating room utilization, it is unlikely to be clinically relevant for patients given that no statistically significant differences in perioperative and pathological outcomes were detected. This finding is consistent with previous reports describing longer operative times during the early implementation phase of the HUGO™ platform [3, 16]. The modular design of the system, including independent arm positioning and docking, may contribute to prolonged setup times and less efficient instrument exchanges compared with integrated robotic platforms. Prior robotic surgical expertise may also modulate outcomes during this early implementation phase: [11] reported that surgeons with prior da Vinci™ experience achieved different perioperative results with the HUGO™ RAS system than surgeons without such experience, underscoring the relevance of operator learning curves when interpreting early comparative outcomes. Both of our surgeons had performed more than 250 da Vinci™ RARPs before the study period, so the HUGO™ learning curve observed here reflects the transfer of established robotic expertise to a new platform rather than the acquisition of robotic skills per se. Operative time decreased by approximately 0.8 min per HUGO™ case, and excluding the first ten cases reduced the unadjusted platform difference by about one third. These findings suggest that part of the operative-time difference reflects the initial learning phase with the HUGO™ system. The fitted learning curve reached the mean operative time of the da Vinci™ cohort (193 min) at approximately the 48th case, i.e., at the end of the study period; however, the mean operative time of cases 11–50 still exceeded that of the da Vinci™ cohort by about 12 min.

The adjusted analyses indicated that the estimated difference in length of hospital stay between robotic platforms was clinically negligible. Both the point estimate and the corresponding confidence interval suggested that any true difference, if present, is unlikely to be of practical clinical relevance.

Although lymph node yield did not differ significantly between robotic platforms, surgeon B retrieved significantly more lymph nodes than surgeon A. The standardized model-based predictions showed an adjusted difference of only 1.4 lymph nodes between the robotic platforms (9.6 vs. 11.0), whereas the adjusted surgeon effect corresponded to approximately five additional lymph nodes. These findings suggest that surgeon-specific factors exert a greater influence on lymph node yield than the choice of robotic platform.

For binary outcomes, including anastomotic integrity, positive surgical margins, postoperative complications, and achieved nerve sparing, adjusted odds ratios were close to unity. However, the corresponding confidence intervals remained relatively wide, indicating that the present study was underpowered to exclude modest differences between platforms. Larger prospective comparative studies are therefore required to obtain more precise estimates of these outcomes.

Interestingly, nerve-sparing surgery was planned significantly less frequently in the HUGO™ cohort than in the da Vinci™ cohort (42.0% vs. 63.2%, *p* = 0.034). In the unadjusted analysis, bilateral nerve preservation was achieved in all patients treated with the HUGO™ system when nerve-sparing surgery had been planned, whereas unilateral or no nerve preservation occurred in 19.4% of patients in the da Vinci™ cohort. This unadjusted subgroup comparison is based on selected patients and should be interpreted with caution rather than as evidence of a technical advantage. After outcome-specific overlap weighting and multivariable adjustment, no statistically significant difference in achieved nerve sparing was observed between the two robotic platforms (OR 1.60, 95% CI 0.44–6.27; *p* = 0.481). These findings suggest that the apparent advantage observed in the unadjusted analysis was largely explained by differences in patient selection rather than by the robotic platform itself. One plausible explanation is that patients in whom technically demanding nerve-sparing surgery was anticipated were preferentially treated using the more established da Vinci™ system during the implementation phase of the HUGO™ platform, whereas straightforward bilateral nerve-sparing procedures, as well as patients in whom nerve sparing was not planned, were treated using both robotic platforms. Prospective comparative studies with standardized indications for nerve-sparing surgery are required to determine whether any true platform-related differences in nerve preservation exist.

The observed positive surgical margin rates of 36% and 39% in the HUGO™ and da Vinci™ cohorts, respectively, were higher than those reported in recent HUGO™ series. In the systematic review by [16] positive margin rates were generally lower, while Gandi et al. reported a rate of 22.2% [10, 16]. A likely explanation is the relatively high proportion of locally advanced tumors in our cohort, with pT3 disease present in approximately half of all patients. Indeed, 32–33% of patients demonstrated pT3a disease and an additional 18–19% demonstrated pT3b disease, indicating a substantially more challenging oncological case mix compared with many published series. This interpretation is further supported by the relatively high proportion of patients classified as high-risk according to the D’Amico classification, particularly in the HUGO™ cohort.

The rate of confirmed anastomotic integrity at the first postoperative cystogram (66% HUGO™, 68% da Vinci™) was lower than the intact-anastomosis rates reported in most contemporary series that use routine, rather than selective, postoperative cystography. In a multi-institutional study of 916 patients undergoing RARP with routine postoperative cystography, [12] reported urinary extravasation in only 6.1% of patients (3.7% minor, 2.4% major), corresponding to a 93.9% negative (intact-appearing) cystogram rate. At our institution, cystography was performed with retrograde instillation of 100 mL contrast medium followed by 100 mL water, with anastomotic integrity confirmed fluoroscopically in two planes both during bladder filling and after voiding; any visible contrast extravasation, however minor, was classified as a non-intact anastomosis. This comparatively strict, two-phase assessment protocol likely accounts for at least part of the discrepancy with series using less stringent or single-phase (filling-only) grading criteria, rather than reflecting a true difference in anastomotic quality — a view supported by the absence of a significant difference in hospital stay or catheterization time between our platforms [12]. A recent systematic review and meta-analysis directly comparing the HUGO™ and da Vinci™ platforms across eight comparative studies (*n* = 1114) likewise found no significant difference in postoperative complications or catheterization time between systems [9], consistent with the absence of a significant platform effect on anastomotic integrity in our adjusted analysis (OR 1.22, 95% CI 0.35–4.36). We therefore consider our anastomotic integrity finding to reflect this stringent, protocol-specific assessment definition rather than a clinically meaningful difference between platforms, and recommend that future comparative studies explicitly report the grading criteria — including whether extravasation is assessed during filling only or also after voiding — used for cystogram-based leak assessment, to enable more direct comparisons across series.

This study had the strength that both surgeons used both platforms so that confounding by personal preferences and skills could be minimized statistically. As shown in Supplementary Table S2, surgeon-related effects were generally small and not statistically significant for most investigated endpoints. The only exception was lymph node yield, which differed between surgeons despite the absence of a platform effect. These findings suggest that surgeon-specific factors may influence selected procedural outcomes independently of the robotic platform and underline the importance of accounting for surgeon effects in comparative studies of robotic surgical systems.

A methodological strength of the present study is the use of outcome-specific propensity score models rather than a single propensity score model for all endpoints. Covariates were selected a priori according to their expected influence on each specific outcome based on clinical relevance and published evidence. Together with overlap weighting and additional covariate adjustment, this approach guarantees covariate balance for the measured covariates included in each propensity score model and provides adjusted treatment effect estimates tailored to each investigated endpoint. This approach is intended to tailor covariate adjustment to the specific confounding structure of each endpoint, which may reduce model misspecification relative to a single shared propensity score model; however, the relative efficiency and bias properties of outcome-specific versus combined propensity score approaches in small samples have not been formally established and warrant further methodological investigation.

Several limitations should be acknowledged. First, the retrospective single-center design introduces the potential for selection bias and unmeasured confounding. Outcome-specific propensity score models were intentionally restricted to clinically relevant preoperative covariates for each endpoint. Given the relatively small sample size, this strategy was chosen to avoid overfitting while preserving clinically meaningful adjustment. Nevertheless, residual confounding by non-included baseline characteristics cannot be completely excluded. Furthermore, a small number of patients exhibited extreme propensity scores (Supplementary Figure S2), indicating limited overlap between treatment groups. By design, these observations received only minimal overlap weights and therefore contributed little to the estimated treatment effects, restricting inference to the overlap population rather than to patients with a near-deterministic treatment allocation. Second, the sample size was relatively small, limiting statistical power to detect modest differences between platforms. Third, several relevant perioperative and functional outcomes, including docking time, instrument exchange time, estimated blood loss, postoperative continence, erectile function, and patient-reported outcomes, were not routinely recorded and therefore could not be analyzed. Because these implementation-specific metrics are central to evaluating the adoption of a new robotic platform, their absence represents an important limitation of this study as an implementation analysis and should be addressed in future prospective work. Furthermore, biochemical recurrence and other long-term oncological endpoints were not available because of the limited duration of follow-up, precluding assessment of oncological efficacy beyond the early postoperative period. As the 50 HUGO™ cases represent the complete clinical HUGO™ experience of both surgeons at our center, this study covers only the early adoption phase; operative times after completion of the learning curve could not be assessed. Cystography timing also differed slightly between platforms as a consequence of differing standard operating weekdays rather than a platform-specific protocol difference (predominantly postoperative day 4 for HUGO™ cases versus postoperative day 5 for da Vinci™ cases); while this reflects routine scheduling, we cannot entirely exclude a modest effect of this timing difference on the observed anastomotic integrity rate. Finally, the follow-up period was insufficient to evaluate long-term oncological and functional outcomes.

Despite these limitations, this study represents one of the first German comparative experiences with the HUGO™ RAS platform in robot-assisted radical prostatectomy. The inclusion of a contemporary da Vinci™ cohort treated by the same experienced surgeons and the application of outcome-specific overlap-weighted regression analyses with additional covariate adjustment strengthen the validity of the findings. Larger prospective multicenter studies with longer follow-up are warranted to further evaluate oncological, functional, and economic outcomes associated with the HUGO™ system.

## Conclusion

In this single-center retrospective comparison, robot-assisted radical prostatectomy performed with the HUGO™ RAS system demonstrated no statistically significant differences in early perioperative and pathological outcomes after outcome-specific propensity score adjustment compared with those achieved with the established da Vinci Xi^®^ platform. Although operative duration tended to be longer during the early adoption phase of the HUGO™ system, no clinically meaningful differences were observed with respect to operative duration, lymph node yield, or length of hospital stay after adjustment. These findings suggest that the HUGO™ platform is a feasible option for robot-assisted radical prostatectomy in experienced robotic centers; however, the limited sample size, selection effects in platform allocation, the early phase of HUGO™ implementation at our institution, and the absence of functional and long-term oncological outcomes preclude any conclusion regarding equivalence to the da Vinci Xi^®^ platform. Larger prospective studies with longer follow-up are required to confirm our results on resection margins, anastomotic integrity, and complications, and to evaluate long-term functional, oncological, and cost-related outcomes.

## Supplementary Information

Below is the link to the electronic supplementary material.

Supplementary Material 1

## Data Availability

The datasets generated and/or analyzed during the current study are not publicly available due to institutional data protection regulations but are available from the corresponding author on reasonable request.
