# Supplementary material for: Implementation of the HUGO™ RAS system for robot-assisted radical prostatectomy: a single-center comparison with the da Vinci Xi® system
Source: World J Urol. 2026 Oct 28;44(1):767. doi: 10.1007/s00345-026-06870-8 (PMC13719753; doi:10.1007/s00345-026-06870-8)
Supplement: Supplementary file 1 [file 345_2026_6870_MOESM1_ESM.docx]

**Implementation of the HUGO™ RAS System for Robot-Assisted Radical Prostatectomy: A Single-Center Comparison with the da Vinci Xi® System**

**Supplementary Material**

Supplementary Table S1: For each outcome, a separate propensity score model was specified using clinically relevant preoperative covariates. Overlap weighting was subsequently applied based on the estimated propensity scores.

| **Outcome** | **Covariates included in the propensity score model** | **Clinical Rationale** |
| --- | --- | --- |
| Operative duration | Surgeon, ASA score, BMI, Planned nerve-sparing | Surgical complexity and patient factors |
| Length of hospital stay | Surgeon, Age, ASA score | Recovery and perioperative risk |
| Anastomotic integrity at first cystogram | Surgeon, Age, BMI, Planned nerve-sparing, D’Amico risk group, Prostate volume | Anastomotic complexity and oncological extent |
| Positive surgical margins | Surgeon, BMI, D’Amico risk group, Prostate volume | Tumor biology and surgical difficulty |
| Lymph node yield | Surgeon, BMI, Age, ASA score, D’Amico risk group | Extent of pelvic lymph node dissection |
| Complications | Surgeon, ASA score, Planned nerve-sparing, Prostate volume, D’Amico risk group | Patient frailty and operative complexity |
| Achieved nerve sparing | Surgeon, D’Amico risk group, Prostate volume, Planned nerve-sparing | Surgical complexity |

Supplementary Figure S1: Outcome-specific Love plots before and after overlap weighting

Supplementary Figure S2: Outcome-specific propensity score distributions by robotic platform

Supplementary Figure S3: Adjusted treatment effects with unadjusted 95%-confidence intervals indicating precision from outcome-specific overlap-weighted regression models

Supplementary Table S2: Estimated platform and surgeon effects across all outcome-specific regression models.

| **Outcome** | **Platform effect** | **Surgeon effect** |
| --- | --- | --- |
| Operative duration | β = +14.6 (-3.0 to 32.2), p=0.104 | β = -7.0 (-25.9 to 11.8), p=0.460 |
| Length of hospital stay | β = -0.003 (-0.022 to 0.015), p=0.720 | β = -0.000 (-0.019 to 0.019), p=0.969 |
| Anastomotic integrity | OR = 1.22 (0.35 to 4.36), p=0.755 | OR = 1.20 (0.30 to 5.00), p=0.793 |
| Positive surgical margins | OR = 0.69 (0.20 to 2.35), p=0.555 | OR = 0.56 (0.15 to 1.97), p=0.374 |
| Lymph node yield | IRR = 0.87 (0.65 to 1.17), p=0.359 | IRR = 1.50 (1.11 to 2.04), p=0.008 |
| Complications | OR = 0.83 (0.18 to 3.60), p=0.803 | OR = 1.05 (0.20 to 5.19), p=0.956 |
| Values represent adjusted effect estimates obtained from the final overlap-weighted outcome models. Platform and surgeon effects were estimated simultaneously within the same multivariable outcome models. β = regression coefficient; OR = odds ratio; IRR = incidence rate ratio; CI = confidence interval. | | |

Supplementary Table S3: Effective sample sizes before and after overlap weighting, standardized mean differences, and minimum and maximum weights (expressed relative to a weight of 1 in an unweighted analysis).

| **Outcome** | **Unadjusted ESS (da Vinci™ / HUGO™)** | **Adjusted ESS (da Vinci™ / HUGO™)** | **Max. \|SMD\| after weighting** | **Min.–max. weight** |
| --- | --- | --- | --- | --- |
| Operative duration | 57.00 / 50.00 | 51.65 / 46.14 | 0.000 | 0.39–1.71 |
| Length of hospital stay | 57.00 / 50.00 | 53.86 / 48.19 | 0.000 | 0.48–1.60 |
| Anastomotic integrity | 57.00 / 50.00 | 51.23 / 45.68 | 0.000 | 0.23–1.83 |
| Positive surgical margins | 57.00 / 50.00 | 52.32 / 46.79 | 0.000 | 0.23–1.67 |
| Lymph node yield | 57.00 / 50.00 | 52.32 / 46.79 | 0.000 | 0.23–1.67 |
| Complications | 57.00 / 50.00 | 50.84 / 45.65 | 0.000 | 0.37–1.73 |
| Achieved nerve sparing | 57.00 / 50.00 | 51.95 / 46.12 | 0.000 | 0.34–1.82 |
| ESS = effective sample size (Kish, 1965); SMD = standardized mean difference. Values are reported separately for each outcome-specific overlap-weighted propensity score model (see Supplementary Table S1 for included covariates). By construction, overlap weighting achieves exact covariate balance (SMD = 0) for all covariates included in each model; effective sample sizes after weighting were reduced by approximately 7–10% relative to the unadjusted sample, reflecting a modest, expected loss of precision rather than an unstable or poorly overlapping weighting solution. Weights are expressed relative to a weight of 1 in an unweighted analysis (n·w/Σw); a value of 2 indicates that a patient contributed twice the weight of an unweighted case. | | | | |

Supplementary Figure S4: Learning curve for HUGO™ RARP. Operative time of the 50 consecutive HUGO™ cases plotted against date of surgery, with linear regression fit (solid line) and 95% confidence band (shaded). By consecutive case number, operative time decreased by 0.79 minutes per case (95% CI -1.62 to 0.04, p = 0.063), corresponding to approximately 39 minutes across the cohort (by date: -0.066 min/day, 95% CI -0.14 to 0.01, p = 0.077).
